# Regulation During the Second Year: Executive Function and Emotion Regulation Links to Joint Attention, Temperament, and Social Vulnerability in a Latin American Sample

**DOI:** 10.3389/fpsyg.2019.01473

**Published:** 2019-07-05

**Authors:** Lucas G. Gago Galvagno, María C. De Grandis, Gonzalo D. Clerici, Alba E. Mustaca, Stephanie E. Miller, Angel M. Elgier

**Affiliations:** ^1^Facultad de Psicología y Relaciones Humanas, Universidad Abierta Interamericana, Buenos Aires, Argentina; ^2^Instituto de Investigaciones en Psicología, Facultad de Psicología, Universidad de Buenos Aires, Buenos Aires, Argentina; ^3^Consejo Nacional de Investigaciones Científicas y Técnicas, Buenos Aires, Argentina; ^4^Department of Psychology, University of Mississippi, Oxford, MS, United States

**Keywords:** executive functions, emotion regulation, joint attention, social vulnerability, temperament, still-face paradigm

## Abstract

Although a growing body of work has established developing regulatory abilities during the second year of life, more work is needed to better understand factors that influence this emerging control. The purpose of the present study was to examine regulation capacities in executive functions (i.e., EF or cognitive control) and emotion regulation (i.e., ER or control focused on modulating negative and sustaining positive emotions) in a Latin American sample, with a focus on how joint attention, social vulnerability, and temperament contribute to performance. Sixty Latin American dyads of mothers and children aged 18 to 24 months completed several EF tasks, a Still-Face Paradigm (SFP) to examine ER (Weinberg et al., 2008), and the Early Social Communication Scale to measure joint attention (Mundy et al., 2003). Parents completed the Early Childhood Behavior Questionnaire Very Short Form to measure temperament (ECBQ-VS, Putnam et al., 2010) and the Social Economic Level Scale (SES) from INDEC (2000). Results revealed the typical responses expected for toddlers of this age in these EF tasks and in the SFP. Also, we found associations between EF and ER and between non-verbal communication related to monitoring infants’ attention to objects (i.e., responding to joint attention) and initiation of pointing (e.g., pointing and showing of an object while the child alternates his gaze to an adult) with EF. Regarding social factors, family differences and type of housing contribute to regulation. For temperament, effortful control was associated with both regulatory capacities. Finally, only age predicted EF. These results suggest that many patterns regarding the development of these abilities are duplicated in the first months of life in a Latin American sample while further highlighting the importance of considering how the environment and the individual characteristics of infants may associate to these regulatory abilities, which is particularly relevant to developing public policies to promote their optimal development.

## Introduction

### EF and ER in the Second Year of Life

Executive functions (i.e., EF or cognitive control) and emotion regulation (i.e., ER or management of emotions) play a fundamental role during child development, contributing to and predicting the development of cognitive abilities, academic achievements, and developmental disorders (Cunningham and Zelazo, 2016; Costa et al., 2017).

The importance of these abilities likely stems from the influence they have on the regulatory capacities. Work during preschool and school age demonstrates that a host of social and individual difference factors relate to these abilities (e.g., Diamond, 2006; Zelazo and Carlson, 2012; Frick et al., 2017). The current research is often limited to primarily Caucasian samples of mid to high socioeconomic status from North America and Europe. Recent work has called for studies of regulation earlier in life to better understand their emergence and the early factors that influence the development of these foundational skills (e.g., Miller and Marcovitch, 2015; Devine et al., 2019).

Although the toddler years have been identified by some as a particularly understudied age in cognitive development (e.g., Hughes and Ensor, 2007). Several frameworks suggest that the development of cognitive control begins early. For example, theoretical EF frameworks suggest the psychological processes involved in the conscious control of thought and action emerge within the second year alongside the emergence of symbols and language. That may aid children in forming task-relevant representations to guide behavior (e.g., Zelazo, 2004;Marcovitch and Zelazo, 2009).

Despite the fact that several researchers have suggested that EF eventually develops to include component abilities such as cognitive flexibility (e.g., changing focus and adapting it to changes in the environment while ignoring the distractors), working memory (e.g., remembering and following directions), and inhibitory control (e.g., stopping impulsive behavior in pursuit of one based on reflection) (Diamond, 2013), other frameworks have highlighted the unity and diversity of EF. These theories suggest that all EF tasks may require something common (i.e., termed a common EF) related to the ability to form and maintain task-relevant information used to guide behavior (Miyake and Friedman, 2012). The proposal of a unitary EF actually aligns well with developmental models showing that unitary models may best explain EF at young ages (Wiebe et al., 2010). This is possibly because developing symbolic abilities can be used to form and maintain information important to a goal. It may underlie an emerging unitary ability in EF (Miller and Marcovitch, 2015).

Empirically, EFs are notoriously difficult to evaluate before the first 3 years. It is necessary to manipulate and sustain meta representations that guide behavior toward the established objective (Zelazo, 2004; Jacques and Marcovitch, 2010). Representational skills in early childhood are still developing. Perhaps the mostly widely measured task thought to tap into early EF is the A-not-B task, in which children have demonstrated the ability to overcome a prepotent motor response, to search for a hidden object at a previously rewarded location and execute search at the correct location over increasing delays with age (e.g., Diamond, 2006, 2013).

However, only recently has research focused on studying early EF with a battery of tasks capable of examining relations between tasks and individual differences. The few studies that have used this approach with infants from 1 to 2 years have shown little consistency and stability in EF skills related to the consolidated ability to guide behaviors through meta representations (Wiebe et al., 2010; Gandolfi et al., 2014; Johansson et al., 2015; Miller and Marcovitch, 2015; Devine et al., 2019). Thus, this has led some researchers to hypothesize that EF may be emerging during the second year of life with component abilities that are not well-integrated yet (Garon et al., 2008; Devine et al., 2019) or not fully developed. This may be supported by emerging representational abilities (e.g., Miller and Marcovitch, 2015).

Unlike EF, ER (i.e., control related specifically to emotion and involves monitoring, evaluating, and modifying emotional reactions to accomplish goals; Thompson, 1994) has a history of early measurement. For example, early measures have often been studied in infants through the Still-Face Paradigm (SFP), which consists of a mother–infant interaction at three phases: (I) mother–infant free interaction in which the dyads are free to engage in a typical interaction with each other, (II) Still-Face period in which the mother stops interacting with the infant and observes them with a “poker” face, and (III) a second mother–infant interaction, in which the mother goes back to free interaction.

Each phase lasts between 90 and 120 s (Stack and Muir, 1990; Mesman et al., 2009) with the critical response focusing on how children regulate emotion, when mothers stop interacting with children in phase II. The typical responses in phase II consist of a decrease in positive affect of the infant (e.g., smiles) and an increase in negative affect (e.g., crying) and aversion to gaze (e.g., avoid looking at the mother) compared to phase I. Further, a carryover effect is also typically observed into phase III, where the type of response produced in the Still-Face phase is continued in phase III, even when the mother re-engages in interaction (Tronick et al., 2005; Gunning et al., 2013).

These trends have been widely observed across cultures (e.g., Ecuador and in ethnic groups belonging to China and Africa; Segal et al., 1995; Kisilevsky et al., 1998; Handal et al., 2017). Examining the critical response to mother’s lack of attention in phase II is indicative of ER, and some have suggested that ER is especially important to study, because learning to modulate negative emotions and sustain positive ones is critical for cognitive development (Kogan and Carter, 1996).

Some researchers have noted an overlap between EF and ER. On the one hand, ER occurs in the service of solving problems and definitions of ER also encompass cognitive and behavioral processes (Carlson and Wang, 2007). On the other hand, to solve classic EF tasks, children need to regulate their emotional reactivity (Miyake et al., 2000; Zelazo and Cunningham, 2007). This has led some researchers to integrate the two constructs by suggesting that domain-general EF may be recruited in the regulation of emotion (Zelazo and Cunningham, 2007).

A distinction has been made between “cool” EF related to abstract problem solving and “hot” EF related to problem solving involving motivation and emotional influences (Zelazo and Carlson, 2012). As EF is typically required in conscious problem solving, when the problem to be solved is ER, EF and ER may be synonymous and more in line with hot EF (e.g., Zelazo and Cunningham, 2007). There is some evidence to support this approach (see Zelazo and Cunningham, 2007) and work with preschoolers demonstrates that individual differences in EF are predictive of ER (Kieras et al., 2005).

However, work examining these relations before 3 years of age are scarce given limitations in studying early EF. Some studies showed that ER and emotion reactivity at 12 and 24 months predicted EFs at 48 and 60 months (Feldman, 2009; Ursache et al., 2013). However, no work to date examines how ER with the SFP may relate to early measures of EF in the second year.

### Joint Attention Links to EF and ER in the Second Year of Life

As previously stated, a number of researchers suggest that representational abilities (e.g., the use of symbols) may be a cornerstone of consciously controlled behavior (e.g., Zelazo, 2004; Zelazo and Cunningham, 2007; Miller and Marcovitch, 2015). Measures of non-verbal communication are often studied during the first years of life when linguistic ability is limited. A significant development during the first months are often studied *via* measures of joint attention; defined as the ability to establish a triad relationship between the infant, an adult, and an object (Bruner, 1975; Tomasello and Farrar, 1986). This type of non-verbal communication is considered fundamental in forming the necessary basis for the subsequent development of linguistic capacity (Tomasello and Farrar, 1986).

Mundy and Gomes (1998) distinguished two types of joint attention: responding to joint attention (RJA, e.g., monitoring the attention of others to objects) and initiating joint attention (IJA, e.g., directing the adult’s attention by pointing or showing). Both dissociate during development, with RJA as a more basic skill linked to a primitive attention system emerging at 6 months, and IJA involving higher levels of attention control emerging at 9 months.

Links between joint attention and ER using the SFP have been established in the first year of life (Weinberg et al., 2008; Mesman et al., 2009). For instance, researchers found that even the number of behaviors related to IJA and coordinated joint increased during the second phase; the SF effect (i.e., decrease in positive affect when mothers disengage) was only associated with RJA measure with the Early Social Communication Scales (Rochat and Striano, 1999; Yazbek and D’Entremont, 2006).

Many researchers (Tomasello and Farrar, 1986; Mundy and Gomes, 1998; Yazbek and D’Entremont, 2006) suggest that RJA is a type of joint attention that does not require volitional attention management system, driven by an understanding of an adult’s intention toward an outside object (e.g., IJA and coordinated joint attention require children’s consciously attempt to engage another). Thus, it is possible that ER as measured by the SFP is related to children’s more passive response to a lack of joint attention bids in their environment, rather than anything more intentional during phase II. However, this work is limited to 1-year-old infants and more research is needed to extend the joint attention–EF relationship in the second year.

Regarding relation between joint attention and EF, there is more evidence that EF and vocabulary develop in parallel, influencing each other dynamically (Fuhs and Day, 2011; Bohlmann et al., 2015; Slot and Von Suchodoletz, 2018). However, the time, strength, and direction of these associations are still under debate and less studied in the early years of life. There is actually a lack of focus on the link between EF and joint attention in early development, likely due to the fact that the study of early EF itself is primarily based on samples of preschool children.

There are few studies that investigate their development in the first years of life (Gandolfi et al., 2014; Mulder et al., 2014; Miller and Marcovitch, 2015; Rodríguez et al., 2017). A few studies that do examine EF and joint attention in the second year find that they do relate in this early period (e.g., joint attention abilities show both concurrent and longitudinal links to EF from 14 to 24 months, Miller and Marcovitch, 2015).

This has been suggested to be linked to the absence and gradual emergence of unified EF, marking the first years as a transition period, during which a unified executive functioning (Wiebe et al., 2010; Miller and Marcovitch, 2015) involves sustained attention and the ability to inhibit external and internal stimuli. This must be developed first—and may be supported by representational abilities in communication—before the formation of more advanced EF abilities in cognitive flexibility and working memory (Marcovitch and Zelazo, 2009; Diamond, 2013). More work examining joint attention links to EF and ER during the second year will be helpful to substantiate theories stressing the importance of early representational abilities to regulatory ones.

### Temperament Links to EF and ER

Another factor thought to influence the development of regulatory abilities are the individual differences children demonstrate in reactivity and self-regulation. They have a constitutional origin due to their genetic endowment, known as temperament (Carranza and González, 2003; Rothbart, 2007). Although the idea of stable individual differences linked to a genetic endowment is a distinguishing characteristic of temperament, it is also thought to be regulated by the environment and learning (Rothbart et al., 2000).

The temperamental styles described by Rothbart (1981) are divided into surgency (positive affect, level of activity, impulsiveness, risk taking), negative affect (fear, anger, sadness, irritability/discomfort), and effortful control (change of attention and focus, perceptual sensitivity, inhibitory control, and activation). Given that work in temperament focused on individual differences present early in development, there is a large body of work focused on infancy and the toddler years (e.g., Rothbart, 1981). However, it is also important to note that effortful control is proposed as later emerging abilities, driven by development in the executive attention network (Rothbart et al., 2000).

Numerous investigations have demonstrated that temperament impacts other measures of regulation during the first years of life (Rothbart and Ahadi, 1994; Rothbart et al., 1994; Lemelin et al., 2006). For example, temperament relates to EF (at preshcool years) and ER (at first year of life), as effortful control promotes the capacity of working memory and inhibitory control, and negative affect decreases it Freund (2018) and Lin et al. (2019). However, results examining temperament links are contradictory (e.g., effortful control or surgency does not always positively influence cognitive performance, Zhou et al., 2012; Yoo and Reeb-Sutherland, 2013; Frick et al., 2017; Lin et al., 2019) and are thus in need of further research.

### Social Vulnerability Links to EF and ER

Finally, an important social factor in the development of regulation is social vulnerability, defined as a multidimensional variable that includes not only the economic income, but also the type of housing, caregiver’s educational level, overcrowding, access to services, stimulation to education at home, and the presence of basic needs. Individuals faced with social vulnerability in Latin America often experience overcrowding, precarious housing, informal work, and incomplete secondary school.

There are a number of studies that have demonstrated an impact of socioeconomic status on EF and ER (Hackman et al., 2015; Lawson et al., 2018). For example, children with unsatisfied basic needs expressed more perseverations in the A-not-B and Tower of London compared to children with satisfied basic needs (Lipina et al., 2004). Also, maternal education and income predicted working memory and planning between age 2 and 3 (Hackman et al., 2015).

However, links between social vulnerability and EF have been only examined in 2-year-old children or older (Hackman et al., 2015; Lawson et al., 2018). Further, a number of these studies are primarily conducted in samples from North America and Europe. Finally, there are few studies examining the impact of SES on the Still-Face task as a measure of ER (Mesman et al., 2009).

### The Present Study

In the present study, we examine regulatory abilities in EF and ER in a Latin American sample of mother–infant dyads during the second year, to address two research objectives: (1) How does EF and ER (as measured by the SFP) develop and relate during the second year? (2) How does joint attention, temperament, and social vulnerability relate to EF and ER?

There are also age and gender differences in cognitive development; we also examine these effects. Specifically, female infants tend to have better performance in different EF and ER tasks (Espy et al., 1999; Lipina et al., 2004). Further, performance across these tasks also typically improves across a short age range. For example, Wiebe et al. (2010) showed evidence that the number of perseverations in the A-not-B and Three Boxes task decreased from 15 and 20 months.

This work is novel in several regards. First, more work is needed to better understand the development of regulatory abilities during the second year, especially given the importance of early intervention to prevent difficulties in these areas (Campbell and Ramey, 1994; Burchinal et al., 2000; Arán-Filippeti and Richaud de Minzi, 2012). Second, a focus on a Latin American sample will corroborate and extend work examining EF and ER links to joint attention, temperament, and social vulnerability to a new sample.

Given that the number of poor people in Latin America reached 186 million in 2016 (30.7% of the population, CEPAL, 2017), it is important to understand how EF and ER develop not only in this novel context but also with regard to socioeconomic status as well. Although this study extends examination of EF and ER to a novel sample, we expected that relations between joint attention, temperament, and SES will align with past research in other populations and older samples.

More specifically, in line with representational theories of EF (Zelazo, 2004), joint attention—especially self-initiated gestures and following others’ attention—should encourage stronger representations used to control thoughts, behavior, and emotions (Zelazo, 2004). Finally, in line with past work, toddlers from households with satisfied basic needs, lower levels of negative affect, and greater effortful control and surgency would have better performance across regulatory tasks in EF and ER.

## Materials and Methods

### Participants

Participants consisted of 60 mother–infant dyads with children from 18 to 24 months, recruited from public and private maternity gardens and homes in the Autonomous City and Province of Buenos Aires, Argentina, sampled using non-probabilistic, intentional, and snowball methods. In the evaluated sample, 38 mothers were Argentine, 15 from Paraguay, 4 from Bolivia, 2 from Peru, and 1 from Ecuador. All the infants evaluated were Argentines.

To find infants with typical development, we screened the clinical histories of both the mother and the child. The selection of the sample followed strict criteria: Spanish as the primary language, normal vision and hearing, no evidence of serious illness, no family history of psychiatric illness, and no history of significant head injuries, seizures, neurological disease, and substance abuse or dependence. Infants did not show symptoms of acute disease and were born full term and with adequate height and weight for gestational age. Three infants were excluded from the final sample because they presented an atypical development (i.e., hearing loss, *n* = 1) and failed to complete the first session because of fussiness (*n* = 2).

### Procedure

Infants were evaluated together with their mothers. Mothers were asked to keep the children in their lap and not to give any kind of help or cues during evaluation. If the children interacted with the caregivers, they were asked to respond in a natural way and direct attention back to the experimenter, so that the evaluations could be continued. Behaviors were videotaped and timed using a Sony HD HDR-CX160^®^ video recorder and a chronometer Model CR202 of the Galileo Italy^®^ line for timing.

Sixty infants were evaluated in EF and joint attention and 50 completed the SFP. The same male evaluator presented the tasks on a table set between the infant and evaluator in the same order to balance fatigue effects across the sample: (a) Object’s spectacle task, (b) Book presentation task, (c) Following-gaze task, (d) A-not-B task with multiple locations, (e) Spatial reversal task, (f) Delay of gratification task, and (g) Face-to-Face Still-Face task. The administration of this battery of tasks took approximately 45 min. After the tasks were completed, mothers answered the SES and temperament measures. For all tasks, two trained observers coded the behaviors of the sessions independently.

### Parent-Report Measures

#### Social Economic Level Scale (SES, INDEC, 2000)

The SES was used to estimate the family socioeconomic level and classify the participants in the standard cutoffs of Unsatisfied Basic Needs (UBN) or Satisfied Basic Needs (SBN) (INDEC, 2000). This scale defined social vulnerability as a multidimensional variable, including the following: (1) paternal and maternal educational level (between 0 and 12 points according to the level of schooling reached), (2) occupational level (between 0 and 12 points according to the type of activity and level of autonomy), (3) housing characteristics (between 0 and 12 points according to the type of household, the type of materials, and access to drinking water), and (4) overcrowding (between 0 and 9 points according to the number of people per room). The maximum total score of the scale was 45 points (total SES).

Children were classified as UBN if one of the following criteria were met: they lived in a precarious settlement (“shantytown”), the house had no bathroom, the house had no access to mains water, it was overcrowded (three or more people per room), elementary school-aged children in the household were not attending school, or the parents in the house did not have a primary school education. The descriptive data for the sample split by socioeconomic status measured *via* the Social Economic Level Scale can be seen in Table 1.

**Table 1:** Composition of the sample by SES and gender.

Groups	Gender		Total
	Male	Female	
UBN	18	17	35
SBN	8	17	25
Total	26	34	60

*UBN, unsatisfied basic needs; SBN, satisfied basic needs.*

#### Early Childhood Behavior Questionnaire Very Short Form (Putnam et al., 2010)

The Early Childhood Behavior Questionnaire Very Short Form (ECBQ-VS) was used to assess temperament in children aged 18 to 36 months, which assesses the emotional behavior of children from the point of view of caregivers. Behavior was classified following a Likert scale of eight points: (1) Never, (2) Almost never, (3) Less than half the time, (4) Approximately half the time, (5) More than half the time, (6) Almost always, (7) Always, and (8) It did not happen.

This test consisted of 36 items that formed three subscales in surgency, negative affect, and effortful control. The effortful control scale evaluated the ability to inhibit or suppress dominant responses. Surgency was related to positive emotion, rapid approach to potential rewards, and high activity level. Finally, negative affect included predisposition to fear, anxiety, sadness, frustration, and discomfort. The Cronbach’s alphas were 0.61 for surgency, 0.65 for negative affect and 0.62 for effortful control. The coefficients are a little lower than those obtained by Putnam et al. (2010) on six samples of children 18–36 months of age. This could be due to the sample size and age (18–24 months) of the present study.

### Executive Function Measures

#### A-Not-B Task With Multiple Hiding Locations (Miller and Marcovitch, 2015)

A box with five holes (9.5 cm in diameter, 7 cm deep) used as hiding places were embedded in a wooden box (43 cm long × 56 cm wide × 7 cm high). The holes were arranged in a semicircle configuration, so that each hiding place was 16 cm from the point where the box would be placed in front of the children to search. Each hiding place was covered by a blue felt that sealed and opened with a Velcro in the middle to reveal the contents of the hiding place. Two white poster boards of 56 cm × 43 cm were also used. The toys presented to children consisted of three small dolls (about 6 cm high). During the familiarization phase, children chose between these three dolls and watched as it was placed in the center hiding location (with the other holes covered by one of the poster boards). Children were then asked to retrieve the doll to become familiar with the instrument and goal of retrieving the hidden object. In the A-trial phase, the toy was hidden in location A in view of the children. A 10-s delay was imposed where the experimenter counted to 10 aloud and all the holes were covered. After the delay, children were asked to search for the object. This procedure for the A-trials was repeated until children found the object three times at location A. Next, children were presented with the B-trials in which the object was moved to a new location, location B. Children were asked to search until they found the object at location B twice.

The hiding locations for the object were counterbalanced and that the center location was not used as a hiding position because it was used during training and children often demonstrate a midline bias to search in the center. Location B was always placed on the opposite side of the midline of location A. Children were considered to search in a location when they broke the Velcro of one of the locations. On B-trials, perseverations (i.e., continued searching in location A) and whether children successfully completed the task (i.e., search correctly twice in B) were measured. This task measured cognitive flexibility (change focus and adapt it to different displacements while ignoring the distractors), working memory (remembering and following directions), and inhibitory control (stopping impulsive behavior in pursuit of one based on reflection).

#### Spatial Reversal Task (Espy et al., 1999)

Two plastic yellow cylinder containers 10 cm in height and 12 cm in diameter were used. They were placed on a blue fabric 30 cm long × 20 cm wide. At the start of each session, the object was hidden under one of the containers (container A) and children were asked to look for the object. Unlike the A-not-B task, children did not observe the hiding of the toy (Pennington and Ozonoff, 1996), as hiding occurred behind a cardboard screen. This was repeated until children found the object four times at container A. Next, the experimenter moved the toy to a new position, container B, and children were asked to continue search until they found the object at location B twice. The number of perseverations (i.e., searches back to container A once the object was moved to container B) and whether children successfully completed the task (i.e., search correctly twice in container B) were measured. This task measured cognitive flexibility (change focus and adapt it to different displacements while ignoring the distractors), working memory (remembering and following directions), and inhibitory control (stopping impulsive behavior in pursuit of one based on reflection to find the object).

#### Snack Delay Task (Kochanska et al., 1998)

A 7-cm-tall yellow bell, a 22-cm-diameter red shallow plastic dish, and a transparent plastic container 14 cm high and 10 cm in diameter were used in this task. Cookies that were sweet with chocolate and vanilla flavor filling of the Mini Oreo^®^ brand of approximately 3 g each were also used. To begin the task, a cookie was placed on a plate and a transparent plastic container was placed on top of it. The experimenter told children that: “you can eat the cookie when the bell rings, you have to wait.” There was a total of three trials, varying in time between 10, 20, and 30 s, respectively. The experimenter measured the average number of trials that children ate the cookie and whether children successfully completed the task (i.e., the children waited until the bell ring to eat the cookie in the three trials). This task measured inhibitory control (stopping impulsive behavior of eating and wait for the bell).

#### Executive Function Reliability Coding

For reliability, the primary coder recorded the measured variables for each EF tasks on all the videos. A second coder recorded the measured variables from 12 randomly selected videos (20% of total). Interrater reliability for continuous variables (intraclass correlation) was significant at the 0.005 level or below and was greater than 0.93 for all EF measures. Reliability for all categorical measures (Kappa) was greater than 0.97.

### Emotion Regulation

#### Still-Face Task (Weinberg et al., 2008)

To assess ER, we used an adaptation of the Still-Face task for children, which, unlike infant measures, is done on the floor and with a series of standardized toys (Weinberg et al., 2008). A children’s play carpet was placed on the floor 120 cm long × 90 cm wide, and three toys were placed on it: a multi-colored ball (20 cm high), a puppet (30 cm high), and a dog plush toy (25 cm high). The toys were kept constant and mothers were told that they could not use other toys external to those presented. The task consisted of three 90-s videotaped phases. In the first phase, the mother had to play freely with children with the provided set of toys. After 90 s, the experimenter moved on to phase II by prompting the mother with a slight sound to cue her to stop playing and observe with a neutral face. It was explained to the mothers that the neutral face involved looking at the child with a poker face and avoiding all contact. In the last phase, the experimenter cued the mother again to resume the phase of free play. The total test lasted 4′30″.

Coding was completed for all three episodes in accordance with the Child and Caregiver Mutual Regulation (CCMR) scoring system (Weinberg et al., 2003). Child affect was measured based on facial expressions (smiles, frowning, etc.) and vocalizations with affective tone (crying, shouting with enthusiasm, etc.). These were divided into two types of affects:

(1) Positive: facial expressions of joy (for example, smile, laugh) and positive vocalizations with exuberance and enthusiasm.
(2) Negative: facial expressions of anger, sadness, fear, subdued/withdrawn, or perplexed/worried affection and negative vocalizations such as crying, complaining, frustrating, irritation, discomfort, or impatience.

In addition, the number of specific behaviors of the children were measured, which included verbal and non-verbal approaches: (a) verbal approach (call the mother by name or role), (b) physical approach (e.g., approaching the mother, touching, or hugging the mother), (c) show the mother a toy (e.g., approach toy or point to an object), (d) aggressive acts (e.g., shouting, throwing a toy, hitting the mother), (e) displacement (time the infant withdraws from the interaction with the mother, leaving the focus of the camera), (f) aversion (e.g., turning the back on the mother), and (g) self-comforting behaviors (e.g., sucking on a thumb or finger). These behaviors were coded according to the frequency (rate per phase), except the duration of the child’s withdrawal from the mother. The behaviors were mutually exclusive. The camera was positioned on a tripod in front of the dyad to get optimal view of the infant face.

#### Emotion Regulation Reliability Coding

For reliability, a primary coder recorded the measured variables in the Still-Face Task for all the videos. A second coder recorded the measured affect from 10 randomly selected videos (20% of total). Reliability for continuous variables (intraclass correlation) were significant at the 0.005 level or below and were greater than 0.80 for all ER measures in the three phases.

### Joint Attention Measures

#### Early Social Communication Scales (ESCS) (Mundy et al., 2003)

The skills of RJA (e.g., follow an adult’s pointing), IJA (e.g., pointing and showing of an object while the child alternate his gaze to the evaluator or the caregiver), and initiation of behavior request (IBR; e.g., behaviors related to the request of an object initiated by the child) were evaluated through the following subscales of the ESCS.

(a) Object spectacle task. Four different objects were presented to children, consisting of a red plastic toy car, a balloon (which varied in color), a rubber toy (which whistles when squeezed), and a rope toy. Objects were presented on three occasions in time periods of 6 s. The toys were positioned out of the reach of the children, and the occurrences of IJA (e.g., child pointing to the object out of reach to initiate shared attention to the object), RJA (e.g., child responding to the experimenter’s pointing to share attention), and IBR (e.g., child pointing or reaching to obtain the object) were measured. If children attempted to initiate joint attention with the experimenter, the experimenter provided them with a brief natural response (e.g., “I see!”). If they requested the toy by attempting to obtain it, the experimenter moved the toy within reach.

(b) Book presentation task. Children were presented with a book with different drawings and textures, called *!‘A comer! of the Tin Cat^®^* edition Guadal for 20 s. This children’s book of 20 cm × 15 cm contained images of different foods and objects (banana, bib, cheese, milk, pear, biscuit, bread, orange, gelatin, and cutlery). Then, the experimenter pointed for 6 s on each page of the book and asked, “What do you see here?” IJA behavior was considered to occur when infants pointed to a picture. IBR was considered to occur if the infant requested to a page of the book by extending his/her arm toward an out of reach object.

(c) Gaze-following task. Four colorful posters were placed to the left, back left, back right, and right of the infant. There were four trials in this task in which the experimenter called children by name and pointed to each poster to determine whether children responded to joint attention bids (RJA). First, the poster on the right was pointed out, then the one on the left, and then the back right and back left. For each trial, the experimenter turned his entire torso, pointed to the poster with a slightly raised elbow off the table, looked at the poster, and said, e.g., “Did you see the doll?” We computed RJA if the child performed the behavior of following the signal, placing his eyes and head in the direction of the object indicated. Infants received credit for IJA behavior if they pointed to the poster to direct the experimenter’s attention before he showed them the posters.

#### Joint Attention Reliability Coding

For reliability, the primary coder recorded instances of IJA, RJA, and IBR for all the videos. A second coder recorded instances of IJA, RJA, and IBR behaviors from 12 randomly selected videos (20% of total). Interrater reliability for continuous variables (intraclass correlation) were significant at the 0.005 level or below and were greater than 0.78 for all joint attention measures.

### Analytic Strategy

To address our first research question focused on examining EF and ER during the second year of life, descriptive statistics are presented in Tables 2, 3. To analyze ER, an analysis of variance (ANOVA) for the intrasubject comparison on the Still-Face Task was made. The application of Bonferroni method was used to compare each phase (I, II, and III). Then, we made a correlation of measures of ER that was presented in Table 4.

**Table 2:** Means and standard deviations of measured variables.

Measures	M (SD)	95% CI	Range	*n*
Executive function				
(1) Perseveration on A-not-B	3.60 (3.96)	[2.40, 4.55]	0–10	60
(2) Perseveration on SR	4.15 (3.43)	[3.02, 4.83]	0–10	60
(3) Eat snack (before sound)	1.28 (1.32)	[0.96, 1.64]	0–3	60
(4) Total EF tasks passed	2.02 (0.95)	[1.74, 2.30]	0–3	60
Joint attention				
(6) RJA	6.94 (2.66)	[6.22, 7.67]	0–9	60
(7) IBR	6.90 (4.59)	[5.77, 8.15]	0–17	60
(8) IJA	11.64 (7.32)	[9.66, 13.62]	0–31	60
Temperament				
(9) Surgency	5.60 (0.70)	[5.42, 5.78]	3.55–7	60
(10) Negative affect	3.37 (0.95)	[3.12, 3.61]	1.91–6.63	60
(11) Effortful control	4.90 (0.76)	[4.66, 5.06]	2.90–6.40	60
Social vulnerability				
(12) Total SES	29.26 (7.74)	[27.26, 31.26]	15–45	60

*SR, spatial reversal; EFs, executive functions; RJA, responding to joint attention; IBR, initiation of behavior request; IJA, initiation of joint attention; SES, socioeconomic status.*

**Table 3:** Comparison of the behavior scores and the different phases in the Still-Face task among infants.

Still-face effect	Phase I	Phase II	Phase III	*F*	*p*	PI–PII	PII–PIII	PI–PIII
	M (SD)	M (SD)	M (SD)					
Positive	2.4 (1.9)	0.2 (0.4)	0.7 (0.9)	26.99	0.000	0.000	0.009	0.000
Negative	0.2 (0.1)	1.4 (1.3)	1.0 (1.2)	14.21	0.000	0.000	0.421	0.001
Show toy	2.5 (2.2)	1.5 (1.6)	1.2 (1.3)	12.33	0.001	0.105	0.812	0.004
Aggressive	0.4 (0.8)	0.7 (1.3)	1.0 (1.1)	4.55	0.095	0.229	1.00	0.065
Physical App.	1.3 (1.3)	2.9 (2.3)	0.9 (1.2)	12.20	0.000	0.006	0.000	0.865
Verbal App.	1.5 (1.5)	2.7 (3.1)	0.8 (1.2)	12.70	0.000	0.024	0.000	0.031
Displacement	11.4 (17.3)	26.3 (25.4)	19.4 (23.1)	9.46	0.000	0.001	0.136	0.067
Aversion	0.2 (0.4)	0.9 (0.8)	1.8 (1.6)	29.28	0.000	0.000	0.033	0.000
Self-comfort	0.2 (0.5)	0.6 (1.2)	0.4 (1.1)	1.89	0.159	0.203	1.00	0.560

*The p-value of the compared Still-Face behaviors in each phase (I, II, and III) was reported. PI, phase I; PII, phase II; PIII, phase III; Physical app., physical approach; Verbal app., verbal approach.*

**Table 4:** Correlations among emotion regulation variables in the SFP (phase II).

Measures	1	2	3	4	5	6	7	8	9
(1) Positive	–	−0.02	−0.13	0.02	0.30^∗^	0.07	0.16	0.08	−0.24
(2) Negative		−	−0.24	0.28	0.46^∗∗^	0.26	0.11	0.45^∗∗^	−0.21
(3) Show toy			−	−0.11	−0.22	0.06	0.12	−0.29	0.18
(4) Aggressive				−	−0.06	−0.02	0.22	0.04	−0.22
(5) Physical App.					−	0.51^∗∗^	−0.37^∗^	0.24	−0.07
(6) Verbal App.						−	−0.43^∗∗^	0.24	−0.09
(7) Displacement							−	−0.05	−0.26
(8) Aversion								−	−0.16
(9) Self-comfort									−

*Pearson correlations are reported for all variables. App, approach. ^∗^p < 0.05; ^∗∗^p < 0.01.*

To address our second research question, a multiple linear regression was performed to assess the ability of the total scores of joint attention scales (RJA, IJA, and IBR), Total SES, Temperament, Gender, and Age to predict EF and ER, in order to determine to what extent the percentage of variance of the scores in this regulation abilities is attributable to these individual and social variables. Finally, Pearson *R* statistic was applied to evaluate the correlation between the quantitative scores of the SES scale (i.e., total education, housing, overcrowding, and occupation) and our temperament measures of interest (i.e., subscale scores for surgency, effortful control, and negative affect) with regulation abilities. In Table 5, we summarized the main correlations of the variables. In all analyses, the probability of a Type 1 error remained at 0.05.

**Table 5:** Correlations among composite measures of regulatory abilities, joint attention skills, SES subdimensions, temperamental styles, and age.

Measures	1	2	3	4	5	6	7	8	9	10	11	12	13	14
(1) Executive functions	–	0.27^∗^	0.31^∗^	−0.05	0.30^∗^	0.38^∗∗^	0.26	0.23	0.33^∗^	0.15	−0.12	−0.15	0.30^∗^	0.28^∗^
(2) Emotion regulation		−	−0.02	0.09	0.12	0.21	0.29^∗^	0.12	0.17	0.20	−0.21	0.05	0.31^∗^	0.01
(3) RJA			−	−0.01	0.37^∗∗^	0.26^∗^	−0.02	−0.05	0.37^∗∗^	0.33^∗∗^	−0.19	−0.17	0.22	0.32^∗^
(4) IBR				−	0.06	0.48^∗∗^	0.36^∗∗^	0.36^∗∗^	0.38^∗∗^	0.17	0.20	−0.11	−0.03	0.28^∗^
(5) IJA					−	0.33^∗^	0.15	0.29^∗^	0.33^∗^	−0.01	−0.20	0.06	0.05	−0.06
(6) Total SES						−	0.79^∗∗^	0.79^∗∗^	0.74^∗∗^	0.61^∗∗^	−0.02	−0.28^∗^	0.18	−0.05
(7) Parent’s education							−	0.74^∗∗^	0.29^∗^	0.27^∗^	0.09	−0.32^∗^	0.03	−0.09
(8) Parent’s occupancy								−	0.28^∗^	0.21	0.16	−0.28^∗^	0.17	−0.19
(9) Housing									−	0.55^∗∗^	−0.04	−0.15	0.24	0.11
(10) Overcrowding										−	−0.02	0.01	−0.07	−0.14
(11) Surgency											−	−0.04	−0.05	0.02
(12) Negative affect												−	−0.31^∗^	0.11
(13) Effortful control													−	0.01
(14) Age (in months)														−

*RJA, responding to joint attention; IBR, initiation of behavior request; IJA, initiation of joint attention; SES, socioeconomic status. Pearson correlations are reported for all variables. ^∗^p < 0.05; ^∗∗^p < 0.01.*

## Results

### EF and ER in the Second Year of Life

#### Executive Functions Task Performance

The means and standard deviations for all major variables are presented in Table 2.

Correlation between performance on the three EF tasks revealed that only perseverative behavior on the A-not-B and spatial reversal were correlated (*r* = 0.53, *p* = 0.001). Number of trials children were unable to delay on the Snack Delay was not related to perseveration on the A-not-B or spatial reversal, *r* = 0.19 and *r* = 0.20, respectively, *p*-values > 0.05. We created a composite score of EF with the average of the number of EF tasks passed.

#### Face-to-Face-Still Face (ER) Task Performance

Descriptive statistics for each still-face effect by phase is presented in Table 3. To examine still-face and carryover effects, multiple repeated-measures ANOVAs were conducted on each effect with phase as the within-subject variable. These tests indicated significant differences by phases (I, II, and III) in the variables of positive affect, negative affect, avoidance, displacement, verbal approach, physical approach, and show toys. No statistically significant differences were found according to the phases in the behaviors self-comfort and aggressive acts.

##### Still-face effects

*Post hoc* analysis using Bonferroni was conducted to examine still-face effects (i.e., a change in behavior when mother’s stopped interacting in phase II). Results revealed a significant increase in negative affect, displacement, aversion to gaze, physical approach, and verbal approach from phase I to phase II (Still-Face). In addition, a significant decrease in positive affect was found.

##### Carryover effects

In addition, *post hoc* analysis was also conducted to examine carryover effects. Results revealed a significant increase in positive affect and aversion to gaze from phase II to phase III. We also found a significant decrease in verbal and physical approach. Finally, we also examined changes from phase I (baseline) to phase III. We found a significant increase in the negative affect, avoidance behaviors, and displacement. There was also a significant decrease in positive affect, show toy, and verbal approach. These results indicate the typical response to the SFP: from phase I to II, there is a decrease in positive affect, and an increase in negative affect and the number of interactions, which continues into phase III. Table 3 summarizes these findings.

##### Relations between still-face effects

Correlation between Still-Face variables during phase II was also analyzed (Table 4). Physical and verbal approach tended to increase and were highly correlated during this phase. As expected, negative affect correlated with the aversion to the gaze. The composite, aggregated score of ER was calculated as the mean of the standardized scores on negative, physical approach, and aversion to gaze during phase II, because these are some of the most important variables in this paradigm and are correlated with each other.

### EF and ER Links to Joint Attention, Temperament, and SES

In Table 5, we summarize the correlations between EF, ER, joint attention, temperament, and SES.

#### Correlations Between EF and ER

We examined the correlation between our two composite measures of EF and ER. Results demonstrated a positive correlation between both measures of regulation (*r* = 0.27, *p* = 0.038), suggesting that the ability to execute cognitive control (EF) is related to the ability to regulate emotions (ER) during the second year.

#### Correlations Between EF and Joint Attention, SES Scale Scores, Temperament, and Age

For joint attention, RJA (*r* = 0.31, *p* = 0.038) and IJA (*r* = 0.30, *p* = 0.044) correlated with EF, with a higher amount of this joint attention behaviors increasing the performance on EF tasks. For SES, composite measures of EF correlated with Total SES (*r* = 0.38, *p* = 0.009) and Total Housing (*r* = 0.33, *p* = 0.030), suggesting that higher socioeconomic status and the quality of the home were associated with better EF. For temperament, composite EF was only correlated with effortful control (*r* = 0.30, *p* = 0.037), demonstrating that children with better effortful control performed better on EF tasks. Finally, for age, a positive correlation was found (*r* = 0.28, *p* = 0.046), with older infants having better performance in EF tasks.

#### Concurrent Predictors of EF

Multiple regression analysis was conducted, examining whether joint attention abilities (IJA, RJA, and IBR), Total SES, Temperament, Age, and Gender predicted composite measures of EF. For EF, the overall model was significant, indicating and explaining 36% of the variability in EF composite scores (*F* = 2.23, *p* = 0.047, *R*^2^ = 0.361). Total SES significantly related to EF (β = 0.419, *p* = 0.045), indicating that as the SES increased, EF abilities across all tasks tended to improve. Age was also significantly related to EF (β = 0.310, *p* = 0.049), indicating older children tended to have higher scores on composite EF. Finally, only RJA predicted composite scores of EF (β = 0.250 *p* = 0.047), indicating that as the capacity of children to follow adults’ pointing and gaze increased, EF abilities across all trials tended to improve.

#### Correlations Between ER and Joint Attention, SES Scale Scores, Temperament, and Age

Composite measures of ER correlated with parent’s education (*r* = 0.29, *p* = 0.039), suggesting that parent’s characteristics are associated with ER behavior in the second year. For temperament, composite measures of ER correlated with effortful control (*r* = 0.31, *p* = 0.019), demonstrating that effortful control increases were related to greater ER.

#### Concurrent Predictors of ER

Multiple regression analysis was conducted examining whether joint attention abilities (IJA, RJA, and IBR), Total SES, Temperament, Age, and Gender predicted composite measures of ER. For this variable, the overall model was not significant (*p* > 0.05). Only effortful control was significantly related to ER (β = 0.319, *p* = 0.036), indicating that effortful control was positively associated with ER.

## Discussion

The present study aimed to examine abilities in regulation during the less studied second year. Results suggested that children from Latin America demonstrate similar patterns of EF and ER performance during the second year. In EF, children appear to demonstrate significant development across this short developmental period. Although children demonstrate individual differences, EF abilities are not well-correlated yet at this age. For ER, the patterns of response in the SFP match those of other countries, as children demonstrate difficulties in ER, in response to a lack of parent attention. Importantly, these abilities in EF and ER also appear to be related in the second year of life. Finally, many of the concurrent predictors of regulation important in older samples (i.e., temperament, and SES) also appear to hold importance during this early developmental period. These results add to the growing body of work suggesting that regulatory abilities are showing significant development during the second year and are already showing links to environmental (e.g., SES) and individual (e.g., temperamental) factors.

### Relations Between Regulation Abilities

Overall performance in both regulation tasks aligned with expectations based on previous work (Weinberg et al., 2008; Miller and Marcovitch, 2015). The typical response to the SFP was observed. These relationships were similar to other works using this paradigm (Mesman et al., 2009). Namely, from phase I to II, there was a decrease in positive affect and an increase in negative affect and the number of interactions—which continues into phase III. This work extends the results of the SFP to a Latin American sample. With regard to EF task performance, results on the search tasks were similar to past research (Espy et al., 1999; Marcovitch and Zelazo, 1999). Although the toddler years are often perceived as a time of struggle with regard to regulation, the majority of the infants solved our EF tasks. The task that proved to be difficult in the present study was the Snack Delay, as few children understood it. This is because of the strongest verbal component as it required the ability to understand vocabulary and the infant is exposed to a highly appetitive reward (Espy et al., 1999). Performance on the Snack Delay task was low in general compared to past work (Kochanska et al., 1998; Kim et al., 2013). There was sufficient variability in performance and no evidence for floor or ceiling effects.

A novel component to our study was the demonstrated link between EF and ER as measured by their response to the SFP. This association could be due to the fact that there is an emotional component that accompanies the development of the EF. More specifically, one of the tasks administered (i.e., the Snack Delay task) is hypothesized to measure more hot EF skills related to regulating more emotional responses directly related to the ER (Zelazo and Carlson, 2012). However, it is also important to note that researchers have also proposed that domain general abilities in EF are likely important to ER (Zelazo and Cunningham, 2007). In this sense, the other two more “cool” tasks likely measure the more domain general cognitive aspect of EF, but for its resolution in this age range, the ability to regulate emotional states is necessary (although to a lesser extent) (Carlson and Wang, 2007). This is some of the earliest evidence to suggest an EF–ER relationship during the second year as measured with the SFP, which is scarce in this age range (due to methodological issues).

### Links to Socioeconomic Status

Environmental variables seemed to play a large role in regulation abilities in our present sample. Infants who came from environments with social vulnerability had less mature performances in EF and ER capacities. This is in line with previous studies suggesting that vulnerable contexts may impact infant regulation *via* adverse effects on brain development. For example, vulnerable environments are associated with higher levels of cortisol in children, which affects the cerebral and cognitive functioning of EF and ER tasks that demand the use of the prefrontal cortex (Arán-Filippeti and Richaud de Minzi, 2012; Doom and Gunnar, 2013). In addition, children from vulnerable contexts are exposed to different environmental toxic agents such as air pollution, less healthy lifestyles, and lower levels of nutrition (which regulate neural development from the prenatal stage, Lipina et al., 2013; Ngure et al., 2014; Kim et al., 2018). Finally, poverty environments impact educational levels and the type of education of the mother and father. This translates to differences in parental styles and levels of sensitivity to interaction—skills that would be associated to EF and ER (Conway et al., 2018; Lawson et al., 2018).

More specifically, the level of education of the caregivers and their type of housing were also associated with these regulation abilities in this age range. It should be noted that the correlation coefficients were between 0.30 and 0.35, comparable or larger than those found in other researches in early childhood (Sohr-Preston et al., 2013; Hughes et al., 2015; Lawson et al., 2018). This suggests that SES explained a portion of the variance in these regulatory behaviors. These skills could be linked to one component of the SES and not others; therefore, more research is required.

However, these results demonstrate the importance of distal context in development. The characteristics of the environment, the type of tasks performed by caregivers, the type of housing, and the educational level would promote cognitive development by emphasizing child stimulation already in this age range. For example, vulnerable environments often bring contexts of stress and violence related to lower levels of effortful control and maternal sensitivity—this has been shown to directly impact child regulation (Vargas-Rubilar and Arán-Filippetti, 2014). The links between lower SES and less mature ER and EF in the present sample align with these studies and extend these links to a younger novel Latin American sample.

### Links to Individual Factors: Joint Attention, Temperament, Gender, and Age

With regard to joint attention, only RJA predicted EF composite measure. This could be due to the fact that a cohesive ability in EF (i.e., consistent positive performance across several EF tasks indicating performance driven by a cohesive cognitive control rather than individual task demands) is hypothesized to emerge in this age range. More specifically, previous studies have shown little consistency and stability in EF skills related to the consolidated ability to guide behaviors through meta representations (Zelazo, 2004; Wiebe et al., 2010; Miller and Marcovitch, 2015). Thus, less mature performance in EF may lead to weak associations between joint attention, with only lower levels of RJA associated with EF. Otherwise said, in the absence of a cohesive ability to control behavior across multiple context, individual task performance may better relate to RJA, which measures children’s sensitivity and response to social cues in the environment.

However, these results are aligned with previous work demonstrating that IJA is associated with the later emergence of a more cohesive EF (Miller and Marcovitch, 2015). RJA, IJA, and EF could be related in this cross-sectional sample since both imply the ability to sustain attention and adapt it to different environmental changes. This also mirrors previous research showing concurrent RJA–EF relationships at 14 and 18 months (Miller and Marcovitch, 2015).

As for temperament, there were correlations between effortful control and the regulatory abilities with coefficients of 0.30, like other researches in this age range (Kochanska et al., 2000; Frick et al., 2017). Effortful control is associated with decreasing impulsive responses, and the maintenance of attention, skills necessary for the correct performance of regulation abilities (Rothbart et al., 2000). This could be because effortful control promotes the capacity of working memory and inhibitory control (Liew, 2012). However, in contrast to other researches (Mundy and Jarrold, 2010; Kim et al., 2014) and similar to others (Zhou et al., 2012; Frick et al., 2017), no associations were found between negative affect and surgency with these regulatory capacities. This may be because temperament styles were not directly assessed but were obtained *via* parental report. More research is necessary to solve these inconsistent results.

Regarding age, associations were also obtained in EF, aligning with the proposal that there is significant development in this regulatory ability during the second year (e.g., Wiebe et al., 2010; Miller and Marcovitch, 2015). Although there were no age differences in ER performance, the absence of associations could be due to the fact that the responses to the SFP are fairly stable throughout the different ages, as the majority of studies have also found a lack of age effects (Mesman et al., 2009; McMahon and Newey, 2018). However, more research is needed using this paradigm at 2 years of age.

### Limitations and Future Directions

Taken together, these results underline the importance of early childhood interventions on individual and environmental variables, and on the incipient relationship between behavioral and emotional components of regulation and EFs and joint attention skills at early stages of development. In addition, it sheds light on the effects of the SFP in a Latin American population, with an effect that is increasingly robust. This reinforces the findings of previous research already mentioned and gives a first approach to the subject in a Latin American population in this age range.

However, our study has several limitations. These include a relatively small sample size, obtained with a non-probabilistic method and limited to a single geographic location (Buenos Aires City and Province). Follow-up studies should aim to overcome these limitations by increasing the size and diversifying the sample. Ours was a cross-sectional study, with all the limitations—and benefits—that such approach affords, and it awaits future longitudinal studies to explore how regulation behaviors unfold within each individual child. These future studies will bring us closer to a better understanding of the role that infant socioeconomic status, non-verbal communication, and temperament play on the development of regulation on the first years of life. Future research should also focus on whether early intervention helps promote the development of these regulation abilities, and it does so disproportionately on those infants with largest socioeconomic need.

Further looking toward the future, our results point toward the potential of early childhood interventions on individual and environmental variables. Researchers might want to focus those interventions not merely on the individual child, but rather on the family unit, with the goal of promoting more effective parenting styles and creating environments more conducive to healthy development.

## Ethics Statement

This study and the protocol were carried out and approved by the Ethics Committee of the University of Buenos Aires. The evaluator explained the tasks to the mothers before they gave written informed consent, both for their own participation and for their child’s participation in accordance with the Declaration of Helsinki.

## Author Contributions

LGG, SM, GC, AM, MD, and AE made substantial contributions to the conception, design, collection, and analysis of the data of the work.

## Conflict of Interest Statement

The authors declare that the research was conducted in the absence of any commercial or financial relationships that could be construed as a potential conflict of interest.
